# Breaking down barriers: Recruiting donors of African ancestry in Ireland

**DOI:** 10.1111/vox.70051

**Published:** 2025-05-23

**Authors:** Allison Waters, Niamh O'Flaherty, Ellen McSweeney, Safa Eltom, Barry Egan, Tor Hervig

**Affiliations:** ^1^ Irish Blood Transfusion Service National Blood Centre Dublin Ireland; ^2^ UCD School of Public Health, Physiotherapy and Sports Science University College Dublin Dublin 4 Ireland; ^3^ UCD National Virus Reference Laboratory University College Dublin Dublin 4 Ireland; ^4^ Accuracy Dublin 6 Ireland; ^5^ University of Bergen Bergen Norway

**Keywords:** African, barrier, blood donation, donor, health promotion, motivation, motivator

## Abstract

**Background and Objectives:**

The diversity of the donor pool has an impact on blood provision for patients with inherited blood disorders, such as sickle cell anaemia. Many patients are critically dependent on red cell transfusions and due to uneven antigen distribution among different ethnic groups, they are at high risk of red cell alloimmunization. The aims of the present study were to understand the motivators and barriers to young Black Irish people donating blood, to explore the views of older Black individuals who were previously excluded but may now be eligible to donate following the recent changes to donor screening, and lastly, to make recommendations for future campaigns that appeal to the Irish Black community.

**Materials and Methods:**

Online focus groups with people of African Ancestry living in Ireland were conducted (*n* = 6 focus groups, *n* = 47 participants). A semi‐structured format was adopted. The focus group transcripts were analysed to identify the key themes expressed.

**Results:**

The main barriers to blood donation were (i) personal beliefs, (ii) distrust of healthcare organizations, (iii) previous negative donation experiences, (iv) a lack of basic information and (v) replacement donation. Motivators to donate blood included (i) altruism, (ii) Black representation, (iii) targeted information, (iv) helping the Black community and (v) building trust.

**Conclusion:**

Blood donor recruitment among the Irish Black community should be informed by an understanding of the varied attitudes to blood donation and the current social norms within Irish African communities. Ireland is a modern multicultural society and its blood donor pool should strive to reflect this diversity.

**Highlights**

- A lack of basic information regarding blood donation is cited as a key barrier to becoming a blood donor for individuals of African ancestry in Ireland.
- Many African countries operate a replacement blood donation system, and the concept of altruistic non‐remunerated blood donation is unfamiliar.
- An explanation about why blood donated from individuals of African ancestry is critically important for supporting those with sickle cell anaemia was a powerful motivator.

## INTRODUCTION

Blood transfusion is a lifesaving therapy for several patient groups. Therefore, a sustainable blood supply is integral to the clinical management of patients, which is highly dependent on effective recruiting and retention of blood donors [1]. In Ireland, the blood donor population is predominantly Caucasian and, like other blood services, is heavily reliant on male donors aged 40 years and over to ensure an adequate national blood supply [2, 3]. However, the lack of diversity within the donor pool has a direct impact on blood provision for inherited blood disorders, such as sickle cell disease (SCD), for which blood transfusion is a critical therapy [4]. Red cell donations from donors of African ancestry often enable better phenotypic matching for patients with SCD, as the blood components are produced from donors of a similar genetic background [5]. In addition, there is a higher prevalence of rare blood types among individuals of African ancestry, making the availability of rare blood types for transfusion in this population more challenging [6]. Thus, due to few donors of African ancestry, individuals with SCD are at increased risk of red blood cell (RBC) alloimmunization [7, 8].

In Ireland, there are an increasing number of patients with inherited blood disorder conditions, and the number of patients with SCD has risen 10‐fold in the last 20 years [2]. The Irish population is estimated at 5.3 million, of which only 5.9% have African Ancestry. In addition, only 1.4% of the current Irish donor population, compared to over 50% of inhabitants with African Ancestry, are estimated to have appropriately matched blood to optimally treat individuals living with SCD. According to the Central Statistics Office of Ireland, most individuals in Ireland with African ancestry are from Nigeria (37%), and of those with African ancestry, approximately 39% were born in Ireland (www.cso.ie). Recent changes to the Irish blood donor qualification criteria and the associated donor infectious disease screening algorithms include the introduction of donor malaria testing, thereby expanding eligibility to donate to a broader range of ethnic groups, particularly those who were previously excluded from donation for having a history of residence in malarious regions [9]. Changes that expand blood donation qualification criteria can minimize the impact of negative word‐of‐mouth on blood donation among ethnic minorities. It is also advised that all donor screening questions should be evidence‐based and removed if they cannot be justified [10]. Although malaria is a risk in many countries, the lack of donor malarial screening had been a specific barrier to the recruitment of blood donors with African ancestry.

The primary motivators to blood donation are reported as altruism, awareness, social obligation, a need to replace blood used and increased self‐esteem. In contrast, the principal barriers reported are fear, inconvenience, apathy, perceived ineligibility and not being directly asked to donate [5, 7]. However, ethnic/racial minorities are under‐represented in blood donor populations in most developed countries [4]. A number of donation systems co‐exist globally, such as voluntary non‐remunerated blood donation, family and replacement donation and paid donation, which may impact blood donation perceptions, depending on cultural background and social norms [6]. Conducting blood drives that are specifically targeted to the Black community has been successfully carried out through outreach and education programmes in the United States and the United Kingdom [7, 11]. Therefore, interventions to increase blood donation among minority communities can be effective and have the added advantage of not only increasing donations but also the ability to match rare blood phenotypes [4]. Despite these successes, only an estimated 8% of donors in the United States are Black, underscoring the need to understand perceptions around blood donation and use this to inform evidence‐based donor recruitment policies [1].

Up until 2023, a person born in a malaria endemic area was excluded from donating blood in Ireland, as well as anyone who has visited malaria endemic areas in the last year, even if born in Ireland. Recent changes to donor qualification criteria and screening included the introduction of malaria testing in 2023, enabling those born in malaria endemic areas to donate, and removal of a question relating to sexual contact with those who were sexually active in HIV endemic areas in 2022. Both of these changes positively impact qualifying donors of African ancestry. The aims of the present study were threefold: firstly, to understand the motivators and barriers to young Irish people of African ancestry donating blood; secondly, to explore the views of older Black individuals who were previously excluded but may now be eligible; and lastly, to make recommendations for future communication campaigns to appeal to the Irish Black community. This study is underpinned by the motivation to understand how the Irish Blood Transfusion Service (IBTS) can effectively appeal to the Irish African community to encourage increased blood donation.

## MATERIALS AND METHODS

### Participant recruitment

Participants were recruited by engaging with the African community in the following ways: (1) through partnership with Africa Centre Ireland and Sickle Cell & Thalassaemia Ireland; (2) at the Ghanaian Independence Day Celebration in 2022; (3) through active recruitment at a Dublin Retail Centre and (4) through open recruitment of the African community. All participants must not have previously donated blood in Ireland. Black ethnicity was defined as either one parent or both parents being of Black African Ancestry.

### Focus group format

**FIGURE 1:**
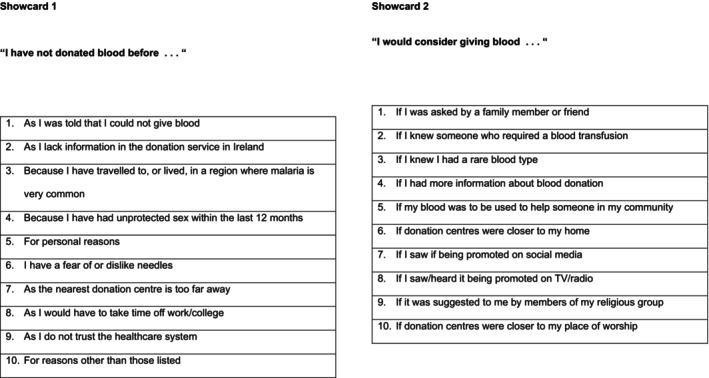
Focus group showcards addressing attitudes to blood donation and healthcare organizations.

**FIGURE 2:**
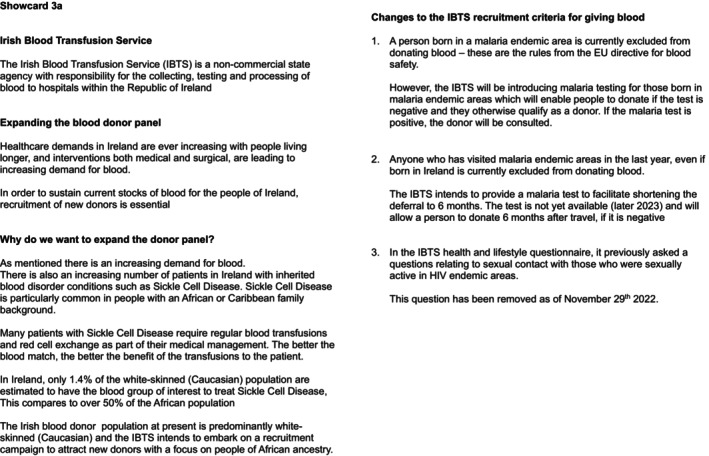
Focus group showcard discussing haemoglobinopathies and their impact on the blood supply.

An online focus group approach was used to facilitate a wide geographic pool of potential participants. A total of six online focus groups with up to eight eligible individuals of African Ancestry were conducted (*n* = 47 participants). A semi‐structured format was adopted. The focus group methodology was informed by the theory of planned behaviour [12] and gathered information on (i) attitudes to blood donation and the IBTS; (ii) the participant's view on their expected blood donation behaviours and norms; and (iii) their individual feelings about blood donation. Briefly, blood donation attitudes and issues (e.g., cultural representation and access) were explored via semi‐structured, open‐ended questions, with prompts and conversation guides to elaborate on topics, as required. The first part of the focus group addressed the current attitudes, motivators and barriers to blood donation, the attitudes to healthcare and its associated organizations, and the attitudes to proposed changes to blood donation qualification criteria. Opinions were informed by any prior knowledge of donation qualification criteria and/or a previous deferral, as well as previous blood donation experiences in Africa and Ireland. The guide materials were used, when necessary, to prompt or direct the focus group conversation (Figure 1). The second part of the focus group specifically addressed the awareness and understanding of haemoglobinopathies, as well as the direct implications of this for the blood supply in Ireland. The guide material specific to this was always used to lead these discussions (Figure 2).

Focus groups, and the associated guide materials, were conducted through, or written in, English and carried out by a specialist company, Accuracy (https://accuracy.ie).

### Focus group participants

Participants were offered a €50 gift voucher to participate in the study and were divided into three main age groups as follows:

1. *Possibility for immediate eligibility to donate*: Individuals, born in Ireland or abroad, aged less than 25 years represent 54% of those in Ireland with African Ancestry. This group may not have travelled to a malaria endemic area recently, nor meet the definition of residency, and have the potential to become ‘lifetime’ donors.
2. *Possibly eligible following recent changes to donor qualification criteria*: Individuals, born in Ireland or abroad, aged 26–44 that were ineligible to donate, but may be eligible following a negative malaria antibody test.
3. *Potential previous negative donation experience*: Black Africans born abroad aged 45–65 years that may have had a negative experience trying to donate blood in Ireland.

The socio‐economic status was categorized as either BC1 (professional, intermediate managerial) or C2D (skilled and semi‐skilled manual workers), based on educational attainment and the occupation of the principal income earner. Other factors considered in designing the profile of the focus groups were the length of time spent in Ireland and cultural background.

### Participant consent

Consent for participation and collection of demographic information was collected prior to the start of the focus group. The focus group's transcripts were de‐identified prior to analyses by the research team. The present study fell outside the scope of the Irish Health Research Regulations and did not require review by a Research Ethics Committee.

### Data collection and analysis

The following data variables were collected from focus group participants: age, gender, socio‐economic status and country of origin or ancestry. The focus group transcripts were analysed manually by a senior qualitative research practitioner, using thematic analysis, to identify the key themes expressed by the focus group participants. The data were coded by theme and, subsequently, by sub‐themes as they emerged (Figure S1). A minimum of three quotes within an age group per sub‐theme was required for it to be considered a valid representation of opinion. Individual quotations from participants were selected to represent the main themes identified (Tables S1 and S2). This analysis was manually verified by a second person. All data were compiled in Microsoft Excel. Lastly, the number of participants that expressed these views was quantified (Table 2)

## RESULTS

### Participant demographics

A total of 47 individuals living in Ireland with African Ancestry participated in the study and had a combined origin or ancestry from 16 different African countries. There were two focus groups per age category, and each broadly comprised an equal mix of men and women. No focus group had more than four respondents from the same country of birth/origin (Table 1). The views expressed and themes identified spanned all age groups and socio‐economic backgrounds. No specific differences between genders or age groups were detected in the views held or attitudes expressed. Work with Sub‐Saharan African communities in Australia has found that single and mixed‐gender focus group discussions yield comparable and robust data when the issue being researched relates to traditional values [13]. Given the large number of African countries represented and difficulties in recruitment, it was not possible to link any attitudes to country of origin or cultural background. However, all views, positive or negative, expressed in relation to the specific themes identified were included in the analyses. Quotes are provided to give context to the themes identified.

**TABLE 1:** Focus group demographics.

Age group	Median age (years)	Male (*n*)	Female (*n*)	Ethnicity as defined by central statistics office	Social class	Focus group format	African countries represented
18–24 years	22	4	4	Black or Black Irish African	BC1	Online	Ghana Nigeria Malawi Uganda Zimbabwe
18–24 years	21	4	4	Black or Black Irish African	C2D	Online	Ghana Kenya Nigeria Mauritius Nigeria Sudan South Africa Zambia
25–44 years	32.5	4	4	Black or Black Irish African	BC1	Online	Benin Ghana Malawi Nigeria Somalia South Africa
25–44 years	29	4	4	Black or Black Irish African	C2D	Online	Congo Kenya Nigeria Sierra Leone South Africa Zimbabwe
45–65 years	50	2	5	Black African born abroad	BC1	Online	Burkina Faso Ghana South Africa Uganda Zambia Zimbabwe
45–65 years	52	4	4	Black African born abroad	C2D	Online	Cameroon Kenya Nigeria
Total		22	25				

### Barriers to blood donation

#### Personal beliefs and concerns

Personal concerns expressed included the fear of needles, uncomfortable with the visual presentations of blood and being unaware of the presence nearby of blood clinics. Many participants referred to being time‐poor and the inconvenience of taking time out to travel to a clinic to donate. ‘I just need time to do it or an easy way to get there’. Religious and spiritual beliefs, regarding giving and accepting blood were a reported issue for some ‘it is more than just a liquid in your blood … there is genetics in there, there is history, the ancestral connections’.

#### Distrust of the healthcare system and organizations

Expressed distrust of the healthcare system, although complex and multifaceted, was a clear barrier to donation and 38.3% (*n* = 18/47) of participants referred to this in their discussions (Table 2). Some participants did not express high regard for the healthcare and medical systems and questioned if the blood service was truly acting for the good of society. This distrust was a combined result of previous negative experiences with hospital visits in their host country, and in Ireland, and a subsequent belief that hospitals should be avoided—‘people know that you have to be in contact with a healthcare facility or healthcare professional if there's something wrong with you.’ Concern surrounding a lack of empathy and understanding of their healthcare needs was also expressed. One participant stated, ‘I think the African perception towards health is very different and it's not as spoken about.’ A total of 29.8% (*n* = 15/47) of participants referenced previous negative experiences with healthcare organizations (Table S1). In addition, a general distrust of the healthcare system, originating for a variety of complex issues, was also a reason not to engage with the IBTS. Previous studies have highlighted that trust in both individuals and the healthcare system predicted willingness to donate in non‐donors from ethnic minorities [14].

**TABLE 2:** Barriers and motivators to recruiting blood donors of African ancestry in Ireland.

Blood donation	Theme	*n*	%
Barriers	Lack of information	33	70.2
	Donor qualification criteria	20	42.6
	Trust in the institution	18	38.3
	Accessibility	16	34.0
	Atmosphere in the donation clinic	16	34.0
	Assumptions of replacement donation	14	29.8
	Negative association with hospitals	14	29.8
	Spiritual/religious beliefs	15	31.9
	Time	8	17.0
	Concern about the effect blood has on donor/patients	7	14.9
	Fear of needles	6	12.8
	Iron deficiency	5	10.6
Motivators	Specific information for Black donors	24	51.1
	Help family or friends, especially if have a rarer blood type that can help	22	46.8
	Emotional connection to the need for blood donors	10	21.3
	Small incentives—easy access, food, time‐in‐lieu from work	10	21.3
	Advertisements	8	17.0
	Social media markets	4	8.5

#### Previous negative donation experiences/deferral

Real and perceived discrimination experienced by African migrants in their everyday social interactions or in institutional settings can act as a barrier to blood donation [13]. There was a strong sense of disappointment among focus group participants (40–65 years), who sought to donate previously but were refused. The view that the previous policy was prejudiced against ethnic minorities was expressed—‘And I did everything in Ireland when I came first, they said to me, oh no, you cannot because you are coming from Africa’. This statements links to another barrier that emerged which was misinformation among the African community that ‘all Africans were excluded from donating blood’. This perception seemed to be grounded in the shared experiences of older African people who sought to donate but were refused. Perceiving oneself as unsuitable for donation, for health, behavioural or ethical reasons is a known barrier tor donation [15]. Concerns about their data protection and the transparency of the process were also raised, ‘IBTS needs to build more trust so that if I do donate blood, I know the blood would go to the right place and help with those numbers essentially’. Some respondents expressed dissatisfaction with the insensitive nature in how they were left waiting for an extended period and felt they were not provided with complete explanations as to why they were refused. Such experiences were described by some as prejudiced, disrespectful, unfeeling and disappointing and one participate felt that ‘a public apology to all the Africans’ *was needed* ‘because I'm not the only one that they've done that to’ (Table S1).

#### Lack of basic information

A lack of basic information regarding the blood donation process was cited as a key barrier (Table 2). Participants reported receiving little to no education about the topic in schools and that blood donation was not a subject discussed with their friends or family. Most participants were unaware of any media campaigns encouraging blood donation. The lack of information created concerns and amplified anxiety about whether (i) it is safe to give blood, (ii) the exact process and (iii) African blood will be accepted. One person stated that they were ‘uncomfortable with the idea, but not because I didn't want to help just cause I didn't know what to expect’. Despite the notable list of barriers, most Africans were supportive of donating blood in Ireland but in the absence of information and encouragement, many lack a clear reason as to why they should donate (Table S1).

#### Replacement donation

Participants reported a low level of blood donation activity in their home country. Many African countries operate a replacement blood donation system and therefore blood donation tended to be associated with a life‐threatening situation, and the concept of altruistic blood donation for the general population was unfamiliar—‘if a family member or friend, someone that's dear to you who's in need, you do anything you can to help them out’. Many participants were aware of SCD but reported that this is often not a subject that is openly talked about among friends and peers. There was a low awareness of the importance of ethnically matching blood donor and recipient (Table S1).

### Motivations to donate blood

#### Altruism

One participant was solely motivated by their religious beliefs or personal values—‘The Koran says if you save a person, one single person, it means that you save the whole world. That's what gives me motivation to give blood’ and ‘I think giving blood is charity, so with any charity, there's not an incentive for you to go volunteer other than knowing that you're helping someone else’ (Table S2). Overall, altruism was a motivator but it needed to be accompanied by targeted information for it to be effective. Culturally adapted health promotion and interventions have been reported to increase blood donation minority communities [16, 17].

#### Black representation

Many reported a lack of targeted communication or advertising to encourage blood donation among the African community. Younger focus group participants reported how a high‐profile social media campaign demonstrating young Black African individuals donating would encourage others to come forward—‘They should show Black influencers or celebrities giving blood’ and to get ‘Influencers involved to help push people of our demographic’. Participants also reported a desire to see more African men and women working in the donation clinics as it would serve to reassure Black donors that they will be treated with dignity and respect, and that the blood service is an open and inclusive organization—‘It should be made sure that there's a committee where people like us or people who are dealing with this are part of those committee. So, our people see who they are, that they look like them’ (Table S2).

#### Targeted information

A consistent issue to emerge across all the groups was the desire for more information regarding the donation process to make an informed choice (Table 2). Many reported a hesitancy to donate in the absence of information to answer their concerns. This opinion was clearly articulated by one participant who after reviewing the information provided on haemoglobinopathies stated that the IBTS should focus on ‘educating us because this will be a new area of information, not just for us, for our children as well’ (Tables S1 and 2). In agreement with previous findings, co‐design of culturally targeted public health campaigns is required to ensure that the messages are acceptable to the Irish African communities [16].

#### Helping the Black community—Kin altruism

Helping a close personal contact was a powerful motivator for donation, and the majority of participants acknowledged a willingness to give blood to help a family member or close friend in need—‘some people might think your blood is your blood kind of thing. Unless, if you have to give to a family member who actually really needed it’. In addition, many reported that if asked by a family member to give blood they would consider it (46.8%, *n* = 22/47). This points to how the desire to help someone close created an urgency that would likely outweigh any concerns about the process of giving blood. In reference to the need for ethnically matched blood, one focus group member stated that ‘It's actually quite shocking to see that most of the wider population can't really help for our community’ and another that ‘I think with that information, if it was more commonplace, it would help serve as a further incentive for people of the African community to donate blood’ (Table S2).

Another more universal theme that emerged across the groups was the busy lives of participants and the inconvenience associated with taking time out of their day to locate and travel to a blood clinic. A convenient place and time to donate is a key motivator for recruiting donors [5, 18, 19], and previous studies have shown that fewer proximal donation opportunities likely contribute the underrepresentation of this community in blood donation [20]. Also, a united national effort should be made by tying donation to a mascot—‘have a cute mascot to get people excited about going to give blood’.

#### Building trust

In order to overcome any scepticism and distrust in the blood service, in particular, among those aged 45–65 years may have had previous negative experience, the focus groups demonstrated a need for community outreach and direct engagement with organizations that have established links with the Irish African community, such as the Africa Centre, the Sickle Cell Society of Ireland and New Communities Partnership. It was clear that ‘proper consultation session where people have the opportunity and the confidence to say, ‘yes’ is needed. Some participants (21%, *n* = 10/47) pointed to the value of a mobile clinic that visited African communities on select days to facilitate people to donate and to how small incentives have proved effective to engage donors. It is known that the risk for donor lapse has been reported to increase with each extra kilometre distance to the nearest donation centre [21]. It was also suggested that blood education could ‘start from the schools and go up to churches because that's where you find most Africans’ (Table S2).

### Blood donation health promotion

#### Attitudes to blood donation

The differences in blood donation systems in Ireland and Africa highlighted that there was limited understanding about the Irish altruistic donation system, and of how this is different the donation processes in place in Africa. This directly impacted the attitudes to donation. One respondent referred to replacement donation and expected to be specifically asked to donate if needed—‘Because in Ghana for example, it's like the only time someone will actually give blood is when they're actually requested by the doctor’. However, concern was raised about the method for targeting donors of African Ancestry and whether when supply was low, would ‘Black’ blood be treated differently—‘I know my blood's going to go back to someone in the African community … if I needed blood transfusion and if there, wasn't say enough blood from someone from Africa or my community, am I not going to get blood’. Again, this points to knowledge gaps in the donation process in Europe, as well as equitable access to the health service in Ireland.

Many of the respondents were familiar with SCD and some were also aware that the condition is more prevalent in the African population. However, the specific information regarding the disease (Figure 2), as well as the low number of Black African people currently donating in Ireland, impacted the respondents, with one individual stating ‘I feel like if Black people knew that then, or even the older generation, if they knew that, they would want to donate blood.’ It has been previously reported that to increase the number of blood donors among African migrants in Australia, promoting knowledge and awareness of issues associated with blood donation, such as the importance of compatible blood for patients of African descent, should be emphasized [8]. Respondents in the present study expressed surprise and alarm following receiving this information and the urgency to acquire appropriately matched blood allowed for a revaluation of their attitudes to giving blood, and a desire to help their fellow Africans—‘I feel like as soon as they mention that part, people are going to be like, oh so we have to get up, go and donate’ and ‘After reading the information, it actually did give me a bit of a motivation to look past my fears, which is the needles and the bag of blood’.

#### Current social norms in the Irish African communities

Focus group participants (40–65 years) who had previously sought to donate but were refused, held a strong sense of disappointment and anger at how they were treated. Some expressed the view that the previous policy was prejudiced against ethnic minorities and took issue with how they were treated in the clinic at the time (Table S1, 10 quotes). Age, blood donation knowledge and country of birth have been previously associated with blood donation in African migrants, but not educational, employment or migration status [8]. Participants in the older age focus group were not opposed to giving blood in the future but remained sceptical regarding about what they perceived as a ‘sudden change in interest’ by the IBTS in the Black African population. One participant felt there was a need for the IBTS ‘to make a public apology to all the Africans’ regarding previous donor acceptance criteria, such as the lack of malaria testing or a donor qualification question pertaining to sex with individuals from Sub‐Saharan Africa.

The recent donor eligibility changes in Ireland were broadly welcomed by focus group participants. However, some of those who were previously excluded still took issue with how they were treated and wondered why it took until 2023 for the introduction of malaria testing. The focus group participants were sensitive to being treated differently to the Caucasian population. One participant asked whether there was ‘going to be an overhaul of the Blood Transfusion Service and all screening processes’. Hence, information explaining that the process of testing people who visit a malaria endemic area is equally applied to all regardless of ethnicity is important.

#### Recruitment

Recommendations for future marketing and engagement campaigns are summarized in Table 3. Each recommended action attempts to address expressed negative attitudes to blood donation, leverage the donation motivators and attempt to normalize blood donation within the African communities living in Ireland. Co‐production and partnership are essential key next steps in recruiting donors of African Ancestry.

**TABLE 3:** Recommendations to support the recruitment of blood donors of African ancestry in Ireland.

Recommendation	Action	Mitigation
Information	Co‐design of literature Specific information describing the needs for ethnically matched blood	Accessible, accurate and targeted blood donation Outline differences in global donation systems
Access	Mobile donation in areas with larger populations of individuals of African ancestry	Misunderstandings surrounding qualification criteria
Staff	Recruitment of non‐White staff	Atmosphere in the donation clinic
Monitoring	Focus groups	Effectiveness of recommendations
Trust	Create donor advocacy group Training for blood serviced staff in empathic interactions with individuals of different ethnicities	Pathway for creating sustainable trust in the National Blood & Tissue Establishment
Marketing	Targeted campaign for Black donors Develop an emotional connection between donors and sickle cell patients in need of blood	Lack of ethnic diversity in the donor pool Overreliance on O negative blood

## DISCUSSION

The present study demonstrated that the provision of targeted blood donation information, as well as positive engagement with the African communities in Ireland, is a crucial step to encourage sustainable blood donation from individuals of African Ancestry. Ireland's closest neighbour, the United Kingdom, has described the prototypical UK blood donor is perceived as White, middle‐aged, middle‐class, college‐level educated and left wing, inferring that blood donation is viewed by many in this area as a White activity. This perception in part explains why ethnic minorities are less likely to donate [22]. Marketing campaigns and literature need to include people of African heritage to help encourage those who may be hesitant about donating.

Receiving information about the donation procedure is a highly ranked motivator among individuals that have never donated before [23, 24]. In addition, increased awareness of the issues associated with blood donation further impacts blood donation decisions [6]. However, information alone is unlikely to be enough to encourage many from the African communities, given the latent distrust in healthcare organizations. Previous studies have demonstrated that tailored interventions for specific cultural or ethnic groups should be underpinned by (1) peripheral inclusion of culturally relevant material; (2) evidence specific to the target group; (3) language that is culturally appropriate and (4) involvement of the community [4, 25]. Therefore, education of the Irish African communities on the need for African donors to assist those with SCD will be a critical component for future recruitment and has potential as a powerful motivator.

The majority of donations in Sub‐Saharan Africa are collected following family recruitment and replacement donation [26]. The present study provides evidence that information surrounding the altruistic donation process in Ireland, as well as reassurance surrounding the availability of blood should it be needed through the national health service, is required to bridge the gap in the understanding of the process and improve recruitment. Critically, in Europe, further studies are needed to explore the balance between the continued support for voluntary non‐remunerated donation and the need to diversify the donor population. Recent evidence has shown that despite a strong commitment to non‐remunerated blood donation, many individuals have indicated that money and other incentives would convince them to donate [27].

The present study provides evidence that should underpin future recruitment campaigns aiming to expand and diversify the Irish donor base. The blood services need to actively demonstrate their intention to diversify donor recruitment through representation of Black individuals in marketing materials, donation information and clinic staff [10]. Deepening ties within the African community is critical to build trust and reassure others that the blood service is acting in the best and public health interest of the wider African population in Ireland. Overcoming perceived discrimination and social exclusion requires sustainable initiatives to increase donation awareness, deepen partnerships and provide demonstrable institutional efforts to address the challenges to blood donation faced by Black citizens [19]. Generic ‘mass’ messages may not resonate with individuals of African ancestry living in Ireland, and links between blood donation intention and whether individuals feel included in their new host society have been previously reported [28]. Previous studies have shown that images on the posters and brochures of representatives of racially and ethnically diverse communities are viewed positively [7]. This should be accompanied by training personnel in donor clinics on the cultural differences of other ethnic groups so that African donors feel welcomed and appreciated.

Experiences of inequitable healthcare and mistrust inform future intentions to engage with the blood services [29]. Distrust of healthcare is rooted in real experiences and is recently reported in qualitative research looking at Black women's perceptions of biospecimen donation for clinical research [30]. Furthermore, public trust and subjective perceptions of the healthcare system within a country, rather than the actual objective state of healthcare, also influence blood donation behaviour in Europe [31].

A number of study limitations must be acknowledged. Firstly, the focus groups were carried out in English and all the materials provided were written in English. Therefore, participants may have been constrained by their English language fluency and certain nuances and interpretations that would be easy to convey through one's first language may not have been possible to express through English. Secondly, the study was initially designed solely for market research purposes. This may have impacted the study design from an academic perspective. Lastly, there were challenges in recruiting participants, further highlighting the current gaps in communication and trust between the IBTS and the Irish African communities.

Overall, the outcomes of this study provide evidence from which to build further research, and support future recruitment, ultimately enabling increased diversity within the Irish donor panel. A sense of urgency and societal emergency can be a powerful motivator [32], and the clinical need for better ethnically matched blood needs to be effectively communicated. In addition, recent publications describing alternative arts‐based approaches as effective in encouraging blood donation [33] may offer complementary pathways to deepen ties with many different communities living in Ireland. Ireland is a modern multicultural society, and its donor panel, and consequently its donor marketing and recruitment strategies, should strive to reflect this diversity.

## CONFLICT OF INTEREST STATEMENT

The authors declare no conflicts of interest. Accuracy was employed to conduct focus groups and perform initial analysis of the results.

## Supporting information


**Table S1:** Barriers to donation.

**Table S2:** Motivators to donations.

**Figure S1:** Thematic Analysis.

## Data Availability

Research data may be made available upon a direct request to https://research@ibts.ie, as appropriate, and within the framework of the General Data Protection Regulations.
