# Supplementary material for: Breaking down barriers: Recruiting donors of African ancestry in Ireland
Source: Vox Sang. 2025 May 23;120(8):765–75. doi: 10.1111/vox.70051 (PMC12390370; doi:10.1111/vox.70051)
Supplement: Supplementary file 1 — Table S1: Barriers to donation. [file VOX-120-765-s001.docx]

**Table S1**

| **Barriers** | **Quote** |
| --- | --- |
| Cultural Beliefs and Concerns | - So, in my culture, it's not against giving blood. But the organ is the issue. |
|  |  |
|  | - When I came into Ireland, I think I came across something if you’re African you can't donate. |
|  | - I was told that Africans doesn't donate blood |
|  | - It is more than just a liquid in your blood. It is, there is genetics in there, there is history, the ancestral connections and all kinds of things |
| Distrust of the Healthcare Systems | - I think the African perception towards health is very different and it's not as spoken about. |
|  | - People know that you have to be in contact with a healthcare facility or healthcare professional if there's something wrong with you. |
|  | - It's not good for women to keep giving blood and because they are probably more prone to being anaemic. |
| Previous Negative Donation Experiences / Deferral | - IBTS needs to build more trust so that if I do donate blood, I know the blood would go to the right place and help with those numbers essentially. |
|  | - What happens to the data that comes from that test? |
|  | - Transparency in terms of what happens with the blood or process. |
|  | - Proper consultation session where people have the opportunity and the confidence to say, yes |
|  | - Is there going to be an overhaul of the Blood Transfusion Service and all screening processes |
|  | - Have to make a public apology to all the Africans because I'm not the only one that they've done that to |
|  | - Malawi is up with malaria as well, so that's what I know. I'm not sure I guess on how people both lived there and still donate as well |
|  | - They're just really, there's this kind of fear that oh my God, what if this happens? What if it's contaminated? What if people get blood, I feel like from a stranger, for them it's a big deal. |
|  | - I tried to in Ireland then there were questions around whether I've lived in a country where there is malaria and I was deemed not suitable for donation so I couldn't donate |
|  | - And I did everything in Ireland when I came first, they said to me, oh no, you cannot because you are coming from Africa, you have malaria, you have this disease and so on. |
| Lack of information | - Uncomfortable with the idea, but not because I didn't want to help just cause I didn't know what to expect I suppose. |
|  | - Just how the procedure goes. I can't really just step out and say I'm going to donate blood today. I'd like to know the ins and outs of it. How I prepare myself? |
|  | - Just how it'll affect them, them so the person I would be donating blood to. If I'm healthy enough to donate blood, how it can affect me? |
|  | - I don't need to hear a sob story. I just need to have time. I just need time. I just need time to do it or an easy way to get there or say you're offering free food or whatever or a chill space cause it's like an hour or something, it's a long period of time. - In order for an African person to do something, there has to be a reason. |
| Replacement donation | - Because in Ghana for example, it's like the only time someone will actually give blood is when they're actually requested by the doctor knowing that your giving blood or something will save this person or family member or something, |
|  | - Some people might think your blood is your blood kind of thing. Unless, if you have to give to a family member who actually really needed it |
|  | - Definitely for me because when if a family member or friend, someone that's dear to you who's in need, you do anything you can to help them out. |
