# Supplementary material for: Breaking down barriers: Recruiting donors of African ancestry in Ireland
Source: Vox Sang. 2025 May 23;120(8):765–75. doi: 10.1111/vox.70051 (PMC12390370; doi:10.1111/vox.70051)
Supplement: Supplementary file 2 — Table S2: Motivators to donations. [file VOX-120-765-s003.docx]

**Table S2: Motivators to Donations**

| **Motivator** | **Quote** |
| --- | --- |
| Altruism | - I think giving blood is charity, so with any charity, there's not an incentive for you to go volunteer other than knowing that you're helping someone else. |
|  | - And the Koran says if you save a person, one single person, it means that you save the whole world. That’s what gives me motivation to give blood |
|  | - If there's a chance that I can save someone else's life, I want that to be possible. So it's kind of like you need to make that personal decision yourself. So, I wouldn't give blood, but if there's a chance that let's say something happens to me, God forbid, if I can save someone else, then yeah, I would do that |
|  | - Actually knowing or being aware that this blood you give actually saved this life or that life. For me, that's more than enough motivation for me to be able to give the blood. |
| Black Representation | - I only see people like the phlebotomists or people that take the blood in the blood banks are like white Irish people. So I would assume that they're just looking to just take blood from white, white Caucasian people. |
|  | - It should be made sure that there's a committee where people like us or people who are dealing with this are part of those committee. So, our people sees who they are, that they look like them. |
|  | - If they're included in committees in mobilisation drives will gain the trust of the African community |
|  | - I think like instead of tying it to people, I’d say tie it to have a mascot, have a cute mascot to get people excited about going to give blood or whatever. |
|  | - Influencers involved to help push people of our demographic |
|  | - They should show black influencers or celebrities giving blood. |
|  | - The Europeans, the UK kind of black people, black people living in Europe, we kind of celebrate Black History month in October. |
|  | - The reason why I'm here today is cause of social media. So, I feel like if you're celebrities or influencers, not even just them, but if everyone was on the move or had a hashtag of oh donate blood or everyone's gassing it open, making it a positive thing, a positive move, everyone would be inclined to donate more |
| Targeted information | - After reading the information, it actually did give me a bit of a motivation to look past my fears, which is the needles and the bag of blood. |
|  | - It's certainly made me want to go and give blood more. So i think it's a very good message. |
|  | - It's actually quite shocking to see that most of the wider population can't really help for our community |
|  | - I knew my blood's going to go back to someone in the African community. I don't think that would be say a big driver per se. Cause then if I saw that and if it was like a big article that it’s going back, then maybe in the back of my mind I'd be like, oh, so if I went into a hospital and it was like if I needed blood transfusion and if there, wasn't say enough blood from someone from Africa or my community, am I not going to get blood, do you get me? So, I would rather it be like, oh you're going to help everyone instead of splitting it. |
|  | - Would always prefer to get one of the ethnic background than get an Irish person. Cause from the experience i've had in the past, they always go better than the Irish. - First thing they can actually focus on is educating us because this will be a new area of information, not just for us, for our children as well. |
| Helping the black community | - So, I think with that information, if it was more commonplace, it would help serve as a further incentive for people of the African community to donate blood. |
|  | - I feel like as soon as they mention that part, people are going to be like, oh so we have to get up, go and donate. |
|  | - I feel like if black people knew that then, or even the older generation, if they knew that, they would want to donate blood, yeah. |
|  | - It is frustrating that we could be helping, especially the people that are even closer to us are going through with all this sickle cell and that is a blood disease, it needs blood transfusion |
| Building Trust | - Maybe a project such as yours will open up dialogue and see what happens there. Then it might be that we come from these ethnic backgrounds, but what about the children that are born here and they don't ascribe to haven't been born in Africa? Haven't lived in Africa and they can contribute. So just being told what is ethnicity - Possibly educating if it could start from the schools and go up to churches because that's where you find most Africans, many people still believe in their religion, so they still attend churches |
