# Supplementary figures and images for: Breaking down barriers: Recruiting donors of African ancestry in Ireland
Source: Vox Sang. 2025 May 23;120(8):765–75. doi: 10.1111/vox.70051 (PMC12390370; doi:10.1111/vox.70051)

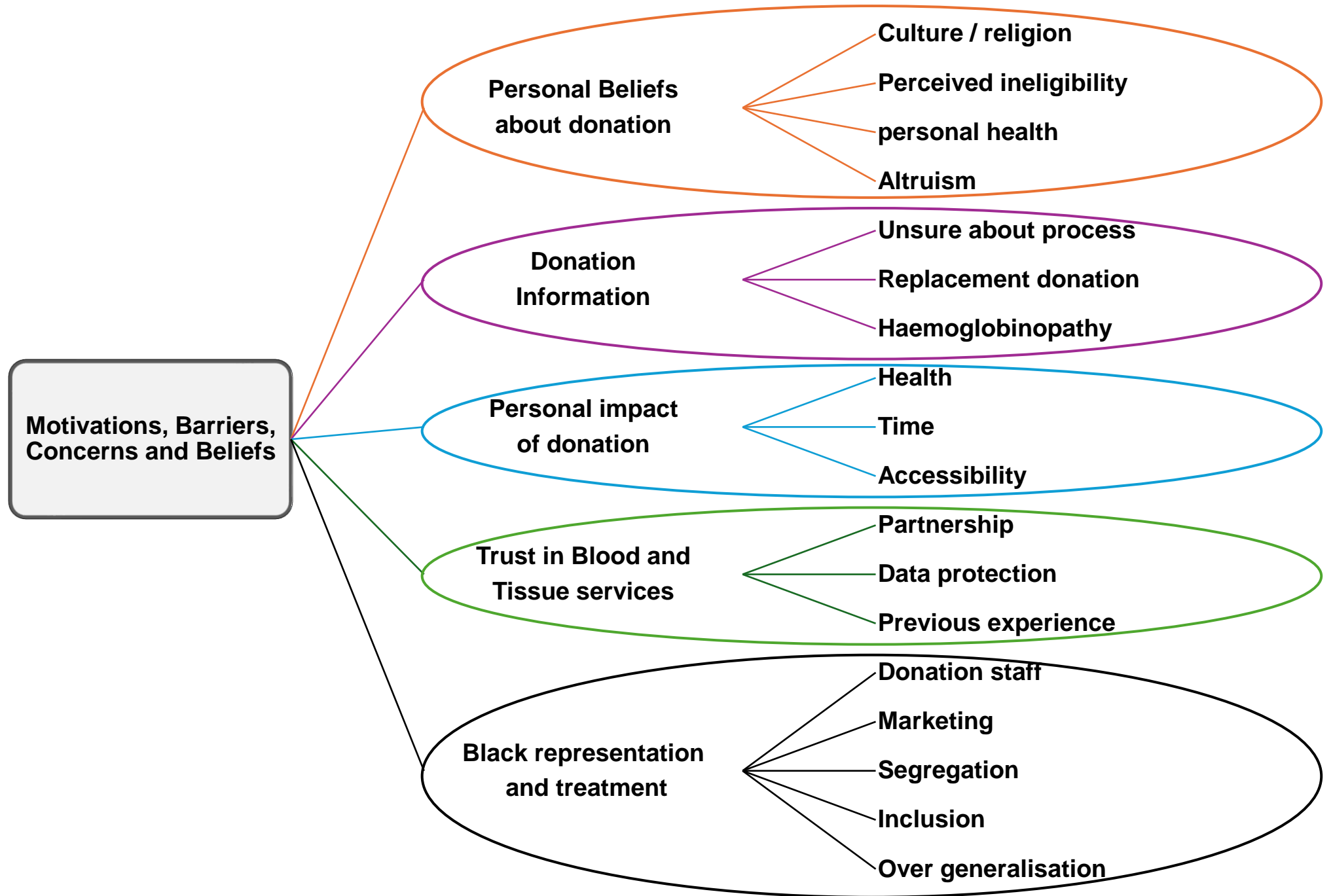

Supplement: Supplementary file 3 — Figure S1: Thematic Analysis. [file VOX-120-765-s002.pdf]
